# A Novel *SACS* Variant Identified in a Chinese Patient: Case Report and Review of the Literature

**DOI:** 10.3389/fneur.2022.845318

**Published:** 2022-03-21

**Authors:** Yuchao Chen, Xiaodong Lu, Yi Jin, Dan Li, Xiaojun Ye, Chenjuan Tao, Menglu Zhou, Haibo Jiang, Hao Yu

**Affiliations:** ^1^Department of Neurology, The Affiliated Hospital of Hangzhou Normal University, Hangzhou, China; ^2^Translational Medicine Center, The Affiliated Hospital of Hangzhou Normal University, Hangzhou, China; ^3^Department of Neurology and Research Center of Neurology in Second Affiliated Hospital, Zhejiang University School of Medicine, Hangzhou, China

**Keywords:** autosomal recessive spastic ataxia of Charlevoix-Saguenay, whole-exome sequencing, novel variant, *SACS*, spastic

## Abstract

Mutations in the *SACS* gene have been linked to autosomal recessive spastic ataxia of Charlevoix Saguenay (ARSACS). It is a clinically and genetically heterogeneous disease characterized by slow progressive ataxia, spasticity, sensorimotor neuropathy, and a combination of other manifestations, such as lack of spasticity, hearing loss, and epileptic seizures. Currently, there have been very few case reports regarding the *SACS* gene mutation in Chinese patients. Here, we describe a 35-year-old Chinese patient carrying a novel variant in *SACS* (c.11486C>T) presenting with progressive ataxia and demyelinating peripheral neuropathy. We then reviewed 22 Chinese cases carrying *SACS* gene mutations, including our patient. All of them had a cerebellar ataxia gait and showed cerebellar atrophy on brain magnetic resonance imaging (MRI). A total of 28 *SACS* mutations were identified in these patients. Our study further expands the mutation spectrum of the *SACS* gene and contributes to the evaluation of genotype-phenotype correlations.

## Introduction

Autosomal recessive spastic ataxia of Charlevoix-Saguenay (ARSACS) is one of the most common autosomal recessive ataxia caused by biallelic mutations within the *SACS* (OMIM: 270550) gene (1). The majority of patients with ARSACS present three core typical phenotypes of early-onset cerebellar ataxia, spasticity, peripheral neuropathy, and other atypical manifestations, including cognition disability, lacking spasticity, epileptic seizures, and hearing loss (2, 3). Brain magnetic resonance imaging (MRI) often revealed remarkable findings of cerebellum atrophy and linear T2 hypointensities in the pons. The optical coherence tomography (OCT) presented a remarkable abnormality in the retinal nerve fiber layer (RNFL) hypertrophy. However, in clinical practice, the absence of remarkable finds in brain MRI or retinal OCT were also present in some ARSACS cases (4, 5).

Genetically, over 200 mutations have been described in the *SACS* gene, most of which have been detected in the gigantic exon 10. The majority of the mutation's types were missense mutation and small deletions subsequently. The identical same mutation leading to different clinical features were described, even in siblings (6). These findings suggested that ARSACS is a clinically and genetically heterogeneous disease and it usually confuses us to make a precise diagnosis. Here, we describe the case of a Chinese patient carrying a novel variant in *SACS* presented with progressive ataxia and demyelinating peripheral neuropathy.

## Case Presentation

**Figure 1:**
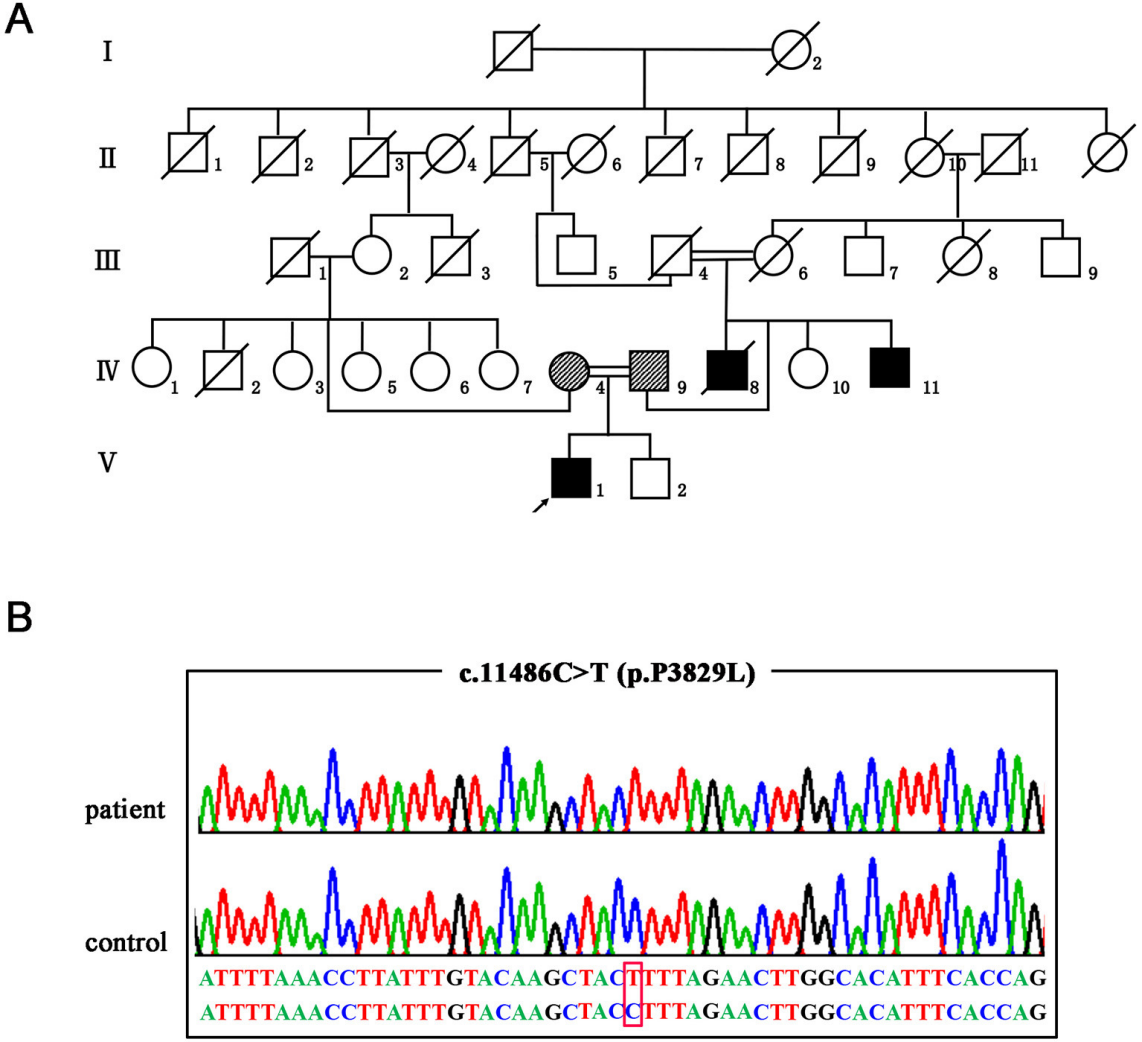
The genetic manifestations of the patient. **(A)** Pedigree of the family. The arrow indicates the proband. **(B)** Validation of the *SACS* mutation (NM_014363: c.11486C>T p.P3829L) by Sanger sequencing.

**Figure 2:**
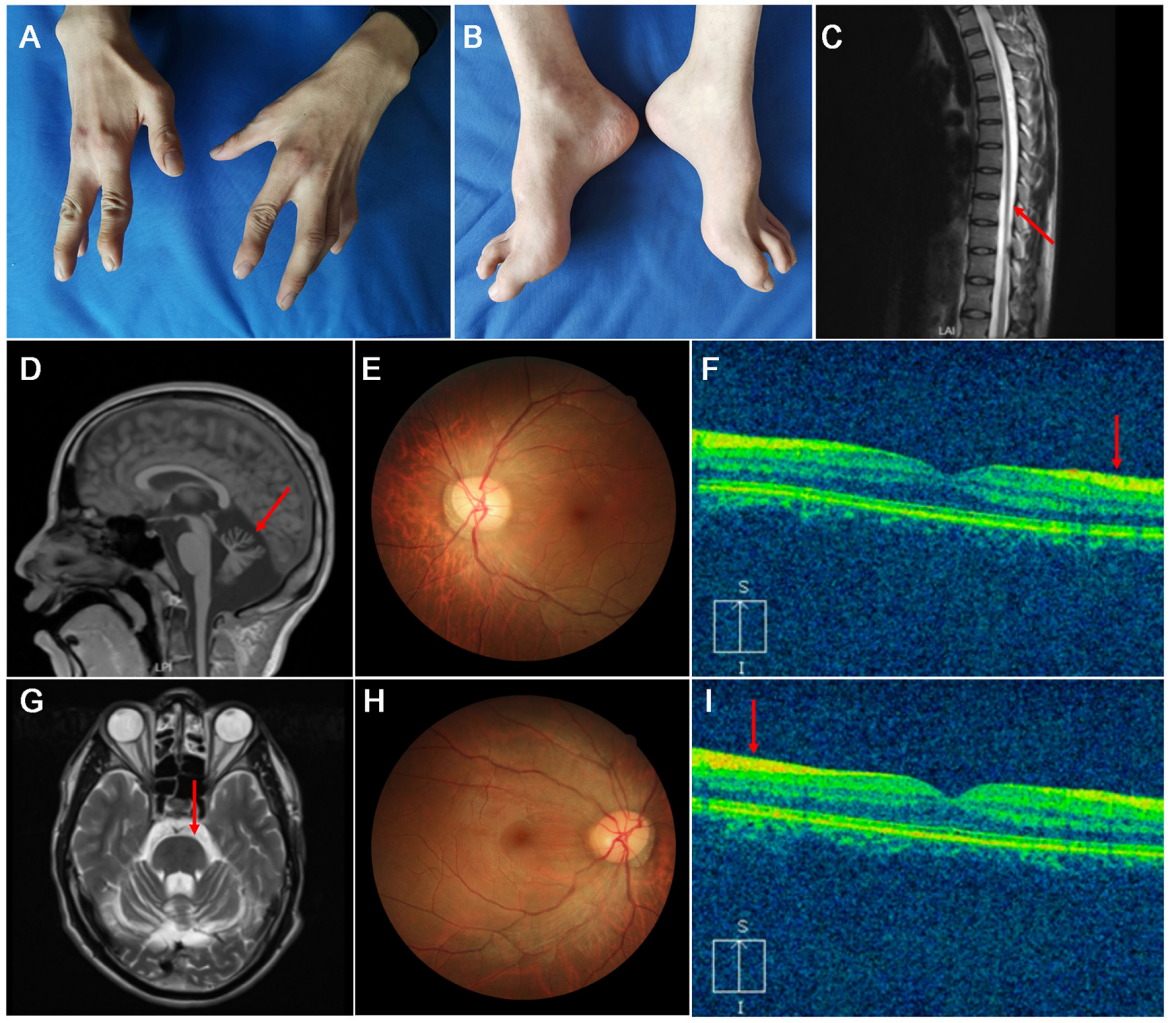
The clinical and neuroimaging features of the patient. **(A,B)** Neurological examinations show Hammer toes and pes cavus. **(C)** Thinning spinal cord in sagittal T2-weighted image (arrow). **(D,G)** The brain MRI shows the cerebellum atrophy in the sagittal T1-weighted image (arrow), hypointensities in the pons in the axial T2-weighted image (arrow). **(E,H)** Fundus photographs of the right and left eyes show swollen papilla with a combed aspect of the interpapillomacular region. **(F,I)** Optical coherence tomography imaging of the right and left eyes shows thickened RNFL (arrow).

The patient is a 35-year-old male from a consanguineous family (Figure 1A). He had delayed developmental motor milestones and began ambulating at 36 months of age. Frequent falls, particularly during running, notably occurred during childhood. He developed a progressive ataxic gait and dysarthria at the age of 28 years. However, with the progression, he needed a mobility aid to protect himself when walking and suffered from dysphagia at the age of 35 years. He did not have a history of seizures, constipation, urinary urgency, or visual problems. His parents did not have any symptoms, but two of his uncles had similar symptoms. The young uncle showed gait problems as a child. These symptoms gradually progressed and resulted in him using a wheelchair at the age of 38. The older uncle died at the age of 59 with similar symptoms. Neurologic examination of cranial nerves revealed significant gaze-evoked nystagmus and moderate dysarthria. Limb examination presented muscular atrophy in lower limbs, and the muscle strength of the distal part of the lower extremities was Medical Research Council (MRC) grade 4. There was decreased tone and tendon reflex in the upper and lower limbs. The sensation examination was symmetric, but the pain sense seemed to be more insensitive in the distal limbs. Extensor plantar reflexes were positive bilaterally. Hammer toes and pes cavus were present (Figures 2A,B). Bilateral finger-to-nose tests, alternate motion tests, and heel-to-shin tests were all awkward. Romberg's sign was positive. The score of the Scale for the Assessment and Rating of Ataxia (SARA) and the International Cooperative Ataxia Rating Scale (ICARS) were 25/40 and 23/100, respectively. The total score of the disease-specific severity index for autosomal recessive spastic ataxia of Charlevoix-Saguenay (DSI-ARSACS) was 23.5, and the clinical Spastic Paraplegia Rating Scale (SPRS) was 22.

Nerve conduction studies (NCS) showed sensorimotor demyelinating polyneuropathy with secondary axonal loss (Supplementary Table 1). A spine MRI revealed thinning spinal cord (Figure 2C). A brain MRI revealed atrophy of the cerebellum on T1-weighted images, and bilateral hypointense stripes in the pons on T2 sequences (Figures 2D,G). Fundus photographs of the eyes demonstrated swollen papilla with a combed aspect of the interpapillomacular region (Figures 2E,H), and OCT depicted hypertrophy in the mild retinal nerve fiber layer(RNFL) (Figures 2F,I).

After genetic counseling, the patient and his parents gave informed consent and the Ethical Committee of the Affiliated Hospital of Hangzhou Normal University in China gave approval. First, we screened causative genes for SCA1, 2, 3, 6, 7, 8, 10, 12, 17, Friedreich's ataxia (FRDA), and Dentatorubral-pallidoluysian atrophy (DRPLA) on the proband. In doing so, we did not identify any pathogenic repeat expansions. We then carried out whole-exome sequencing (WES) and detected a novel homozygous missense variant in the *SACS* gene (NM_014363.5: c.11486C>T p.P3829L). Afterward, segregation analysis by Sanger sequencing confirmed that the patient's parents were heterozygous carriers and his affected young uncle was also homozygote (Figure 1B). No other known pathogenic variants were identified in the WES study. The variant of c.11486C>T in the *SACS* gene was absent in databases of dbSNP, gnomAD, and ExAC. SIFT, Polyphen-2, Mutationtaster, and CADD all predicted that the novel missense variant was deleterious. According to the American College of Medical Genetics and Genomics (ACMG), the variant c.11486C>T within *SACS* is a variant of likely pathogenic (PM2, PP1_Moderate, PP3, PP4).

**Figure 3:**
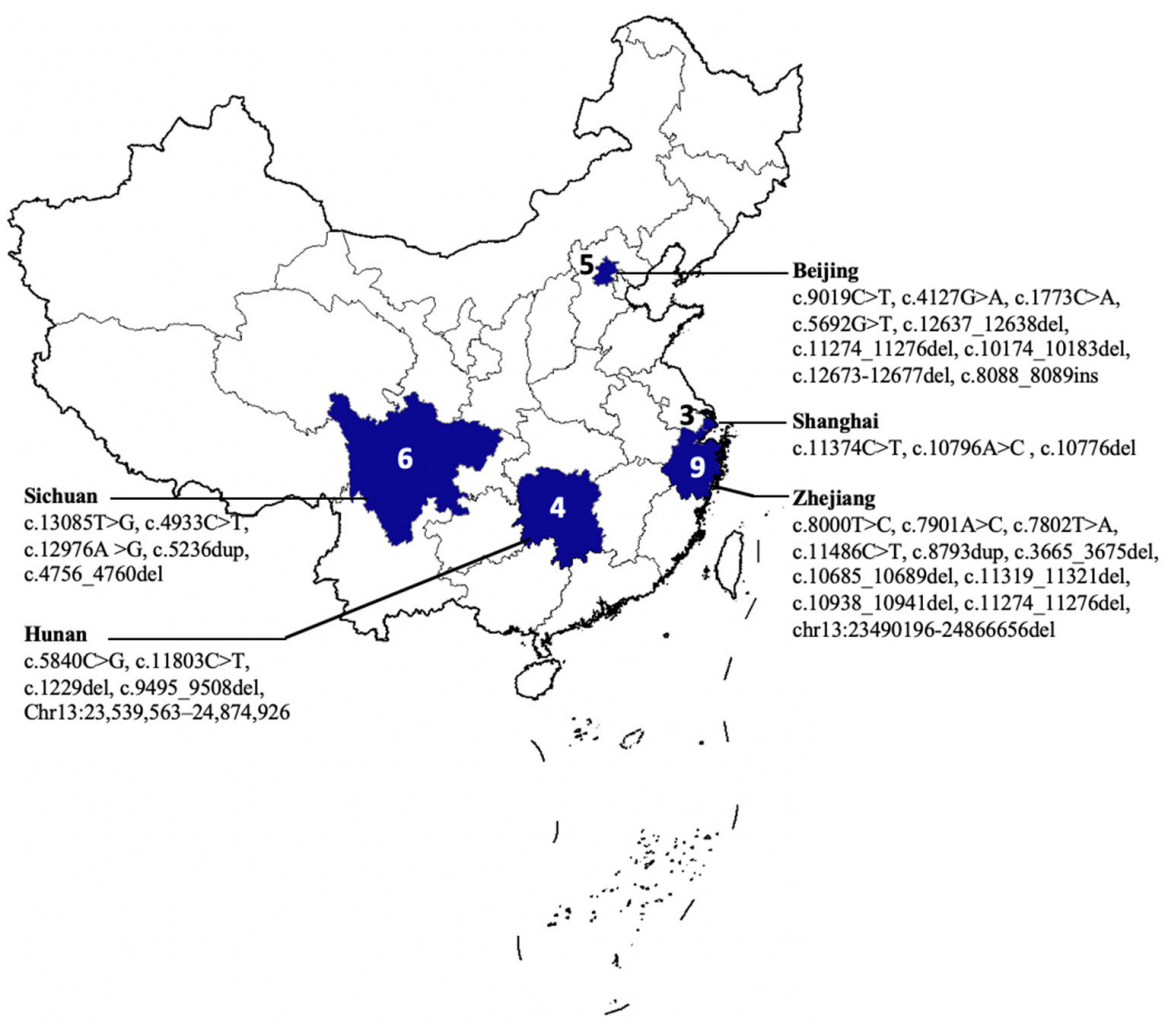
The geographical distribution of patients with autosomal recessive spastic ataxia of Charlevoix Saguenay (ARSACS).

We summarized the clinical and genetic features of our case and the reported Chinese patients (Table 1) (2–4, 7–17). The majority of the families were from Southeastern China (Figure 3). Among them, 16 were men and 11 were women. The age of disease onset in the cases (77.7%, 21/27) was no more than 6 years. All of the 27 cases had onset with ataxia, and 15 of them had spastic gait, whereas three cases showed an absence of spasticity, 16 cases showed pes cavus, and 21 patients presented peripheral neuropathy. Almost all of them revealed cerebellar atrophy on a brain MRI, except for one case, due to his young age (3 years). In 15 patients, the MRI displayed signal hypointensities within the pons, and eight cases showed thickening RNFL. Genetically, 12 of 27 cases carried homozygous mutations in the *SACS* gene. A total of 35 *SACS* gene mutations were identified in the Chinese patients, including 10 missenses, seven non-senses, 16 small deletions, and two gross deletions. Except for two gross deletions, only one mutation was located in exon 8, while almost all mutations were identified in exon 10 of the *SACS* gene.

**Table 1:** Clinical data of Chinese autosomal recessive spastic ataxia of Charlevoix Saguenay (ARSACS) cases from the present study and other related published studies.

**Reference or patient**	**Gender/ Age**	**AAO (years)**	**Ataxia**	**Spastic gait**	**Dysarthria**	**Nystagmus**	**Babinski's sign**	**Pes cavus**	**cerebellar atrophy on MRI**	**Spinal cord atrophy**	**Signal hypointen- sities within the pons**	**Thicken- ing RNFL**	**Periphe- ral neuropathy**	**Others**	**Nucleot- ide mutat- ion 1**	**Nucleot- ide mutat- ion 2**
Chen et al. (7)	M/39	1	+	+	+	+	NA	NA	+	NA	NA	NA	NA	NA	c.1229 delT	c.5840 C>G
Chen et al. (7)	F/35	1	+	+	+	+	NA	NA	+	NA	NA	NA	NA	NA	c.1229 delT	c.5840 C>G
Liu et al. (8)	F/12	6	+	+	NA	+	+	+	+	+	+	+	+	NA	c.11803 C>T	Chr13: 23,539, 563–24, 874,926
Zeng et al. (9)	F/34	27	+	NA	NA	NA	-	NA	+	NA	NA	NA	NA	NA	c.949 5_950 8delTT TTGATG CAAAAC	c.949 5_950 8delTTTT GATGCAA AAC
Sun et al. (4)	M/26	13	+	NA	-	NA	+	+	+	+	+	+	+	NA	c.126 37_126 38delGA	c.1127 4_112 76delAAC
Li et al. (10)	F/21	3	+	+	+	+	+	+	+	+	NA	NA	+	Epilepsy	c.5236 dupA	c.1308 5T>G
Li et al. (10)	M/10	<3	+	+	NA	+	NA	NA	+	+	NA	NA	+	NA	c.523 6dupA	c.1308 5T>G
Zhang et al. (2)	F/22	<3	+	-	-	NA	+	NA	+	NA	NA	-	NA	Cognitive impairment	c.3665_ 3675de lGTGCTG TCTTA	c.3665_ 3675del GTGCTG TCTTA
Guan et al. (11)	M/16	1–2	+	+	+	+	+	+	+	NA	+	+	+	NA	c.1137 4C>T	c.1137 4C>T
Guan et al. (11)	F/17	<3	+	+	+	+	NA	+	+	NA	+	+	NA	Bullae of lung	c.113 74C>T	c.113 74C>T
Lu et al. (12)	M/14	2	+	NA	NA	+	+	+	+	NA	+	NA	+	PKD	c.901 9C>T	c.1017 4_1018 3delGTA AAGATAC
Lu et al. (12)	F/12	1.5	+	NA	+	+	+	NA	+	NA	NA	NA	+	PKD	c.412 7G>A	c.412 7G>A
Jiao et al. (13)	M/35	27	+	+	+	+	+	+	+	NA	NA	NA	+	NA	c.493 3C>T	c.493 3C>T
Jiao et al. (13)	F/37	3	+	+	+	+	+	+	+	NA	NA	NA	+	Cognitive impair- ment	c.1297 6A>G	c.1297 6A>G
Wang et al. (14)	F/36	3	+	NA	+	+	+	+	+	+	+	+	NA	Epilepsy? UIU	c.177 3C>A	c.808 8_8089i nsCA
Wang et al. (14)	M/9	6	+	NA	+	+	-	NA	+	NA	+	NA	+	NA	c.569 2G>T	c.1267 3-12677d elTATCA
Chen et al. (15)	M/30	<3	+	-	+	NA	+	+	+	NA	+	NA	+	NA	c.106 85_10 689de lTCTTT	c.800 0T>C
Cheng et al. (3)	M/3	1	+	NA	-	-	NA	NA	-	NA	+	NA	+	Abnor- mality of the dentition	c.1093 8_1094 1delAGAA	chr13: 2349019 624866 656del
Cheng et al. (3)	M/31	1	+	+	+	+	NA	+	+	NA	+	NA	+	Hearing loss of right ear, mild intellectual disability, muscle atrophy all limbs	c.800 0T>C	c.106 85_106 89delT CTTT
Cheng et al. (3)	F/21	1	+	+	+	-	NA	+	+	NA	+	NA	+	-	c.879 3dupA	c.879 3dupA
Cheng et al. (3)	M/26	4	+	+	+	+	NA	+	+	NA	+	+	+	Epilepsy	c.790 1A>C	c.113 19_1132 1delCTT
Cheng et al. (3)	M/37	11	+	NA	+	+	NA	+	+	NA	+	NA	+	Hearing loss of left ear	c.780 2T>A	c.780 2T>A
Cheng et al. (3)	M/46	39	+	NA	-	-	NA	NA	+	NA	+	NA	+	Weakness of limbs, muscle atrophy of lower limb	c.1127 4_1127 6delAAC	c.1127 4_1127 6delAAC
Chen et al. (16)	F/21	3	+	+	NA	+	+	+	+	+	NA	NA	+	Epilepsy	c.1308 5T>G	c.523 6dupA
Chen et al. (16)	M/23	1	+	+	NA	NA	+	NA	+	NA	NA	NA	+	NA	c.475 6_4760de lAATCA	c.475 6_4760de lAATCA
Zhou et al. (17)	M/34	23	+	+	-	-	+	-	-	+	-	+	+	NA	c.1077 6delA	c.1077 6delA
**Present study**	M/35	<3	+	-	+	-	+	+	+	+	+	+	+	-	c.1148 6C>T	c.114 86C>T

*AAO, Age at Onset; NA, not available, (+), indicates the presence of a feature in an affected subject; (-), indicates absence of a feature in an affected subject; M, male; F, Female; (+), positive; (-), negative; RNFL, Retinal Nerve Fiber Layer; PKD, Paroxysmal Kinesigenic Dyskinesia; UIU, Urge Incontinence Urine*.

## Discussion

Currently, there have been very few case reports regarding *SACS* gene mutation in Chinese patients. In this study, we presented a Chinese ARSACS patient exhibiting early-onset cerebellar ataxia, pyramidal signs, and peripheral neuropathy. By performing a WES and segregation analysis, we identified a novel variant c.11486C>T mutation at the homozygous state in the *SACS* gene.

Different from the clinical features of progressive spasticity and preserving tendon reflexes throughout the disease course in Quebec patients (1, 18), our index case lacked the signs of leg spasticity and decreased tendon reflexes. Additionally, a few ARSACS cases without spasticity in the lower limbs were also observed in different racial groups (19, 20). This finding is in line with other previous studies of patients with ARSACS. There is a possibility that the presence of severe neuropathy with demyelinating features could mask any spasticity. However, there was one ARSACS case in the previous reports, which presented neither spasticity nor neuropathy (6).

The lacking-spasticity phenotype may be associated with the localization of the *SACS* mutation. In our patient, this new variant (c.11486C>T p.P3829L) was located downstream of the UBE3A binding domain (UBD) in the C-terminal of the sacsin protein. The UBD domain may interact with the ubiquitin ligase Ube3A, acting as an important role in hereditary spastic paraplegia (HSP) (21, 22). The patients carrying *SACS* mutations in the UBD domain usually showed leg spasticity and obvious features of ataxia (21). However, another two patients harboring the homozygous variant (c.11542_11544del) located downstream of the UBD domain presented with ataxia without spasticity (6). Together, the *SACS* variants located downstream may not affect the UBD domain's function. Additional functional studies are needed in order to confirm the role of those domains at the C-terminus in the *SACS* gene in protein.

## Conclusion

Collectively, we reported a Chinese ARSACS case carrying a novel variant in *SACS*. Our study further expands the mutation spectrum of *SACS* and contributes to the evaluation of genotype-phenotype correlations.

## Data Availability Statement

The datasets presented in this article are not readily available due to ethical and privacy restrictions. Requests to access the datasets should be directed to the corresponding author.

## Ethics Statement

The studies involving human participants were reviewed and approved by the Ethical Committee of the Affiliated Hospital of Hangzhou Normal University. The patients/participants provided their written informed consent to participate in this study. Written informed consent was obtained from the individual(s) for the publication of any potentially identifiable images or data included in this article.

## Author Contributions

YC, XL, YJ, DL, XY, CT, MZ, HJ, and HY initiated the project and collected and analyzed the data. YC wrote the manuscript. HY commented on and revised the manuscript and supervised all aspects of the project. All the authors read and approved the final manuscript.

## Funding

This study was supported by the Medical and Health Science and Technology Project of Zhejiang Province to YC (2021KY898).

## Conflict of Interest

The authors declare that the research was conducted in the absence of any commercial or financial relationships that could be construed as a potential conflict of interest.

## Publisher's Note

All claims expressed in this article are solely those of the authors and do not necessarily represent those of their affiliated organizations, or those of the publisher, the editors and the reviewers. Any product that may be evaluated in this article, or claim that may be made by its manufacturer, is not guaranteed or endorsed by the publisher.
