# Supplementary material for: A Novel SACS Variant Identified in a Chinese Patient: Case Report and Review of the Literature
Source: Front Neurol. 2022 Mar 21;13:845318. doi: 10.3389/fneur.2022.845318 (PMC8978317; doi:10.3389/fneur.2022.845318)
Supplement: Supplementary file 1 [file Table_1.DOCX]

**Supplementary table 1 Results of nerve conduction studies in the proband with ARSACS**

| **Upper - limb nerves** | | **DML (ms)** | **MCV (m/s)** | **Distal CMAP (mv)** | **Proximal CMAP (mv)** | **SNAP (μV)** |
| --- | --- | --- | --- | --- | --- | --- |
| **Median nerve** | left | 5.27 | 39.4 | 10.1 | 10.2 | NR |
|  | right | 4.79 | 34.6 | 5.9 | 4.9 | NR |
| **Ulnar nerve** | left | 4.34 | 37.9 | 5.1 | 4.3 | NR |
|  | right | 3.66 | 35.5 | 8.1 | 8.6 | NR |
| **Lower - limb nerves** | |  |  |  |  |  |
| **Peroneal  nerve** | left | NR | NR | NR | NR | NR |
|  | right | NR | NR | NR | NR | NR |
| **Tibial nerve** | left | NR | NR | NR | NR | NR |
|  | right | NR | NR | NR | NR | NR |

**Abbreviations:** NR: No Response; DML: Distal Motor Latency; MCV: Motor Conduction Velocity;

CMAP: Compound Muscle Action Potential; SNAP: Sensory Nerve Action Potential; SCV: Sensory

Nerve Conduction Velocity.
